# Kangshujiangu granules improve NAFLD via cross-organ autophagy activation shared with osteoporosis

**DOI:** 10.3389/fphar.2025.1633813

**Published:** 2025-08-14

**Authors:** Ningxi Wang, Li Song, Yue Peng, Langqiu Wei, Zhaofan Long, Che Wang, Qiling Liu, Chuandao Shi

**Affiliations:** ^1^ School of Public Health, Shaanxi University of Chinese Medicine, Xianyang, China; ^2^ Guangzhou Institute of Biomedicine and Health, Chinese Academy of Sciences, Guangzhou, China

**Keywords:** autophagy, AMPK/mTOR/ULK1 pathway, Kangshujiangu granules, non-alcoholic fatty liver disease, osteoporosis

## Abstract

**Background:**

Osteoporosis (OP), an age-related skeletal disorder characterized by reduced bone mass and deteriorated microarchitecture, and non-alcoholic fatty liver disease (NAFLD), a metabolic condition primarily driven by obesity and insulin resistance (with age as a modifying factor), were investigated in this study. We aimed to elucidate the correlation between OP and NAFLD-associated lipid metabolism, and determine the therapeutic effects and molecular mechanisms of Kangshujiangu granules (KSJG) on NAFLD pathogenesis.

**Methods:**

Clinical study: 261 patients were stratified by OP T-scores into OP and non-OP groups. Bone density, lipid profiles, and liver function were analyzed for NAFLD-OP correlations. *In vivo*: 60 female SD rats were randomized into control, NAFLD-OP model, low/medium/high-dose KSJG, and Icariin (ICA) groups. Metabolic indicators and autophagy related proteins were evaluated. *In vitro*: FFA-induced hepatocytes were treated with Compound C, KSJG, or FF. Cells were divided into six groups: BSA control, FFA model, FFA + Compound C, FFA + KSJG, FFA + FF, and FFA + KSJG + Compound C. Lipid accumulation, metabolic markers, and autophagy proteins were analyzed.

**Results:**

Clinical findings: Osteoporosis prevalence exhibited age-dependent elevation. Age, BMI, TC, and TG as independent risk factors for OP (*P* < 0.05). *In vivo* results: KSJG granules significantly attenuated body weight, reduce liver wet weight and liver index; improve lipid accumulation and lipid metabolism disorders; increase autophagy levels in liver tissue, reduce the expression of p-Akt, p-mTOR and p-ULK1 (Ser757), and increase the expression of p-AMPK and p-ULK1 (Ser555) (*P* < 0.05). *In vitro* experiments:Both KSJG and FF significantly reduced FFA-induced TG, ALT, AST, and lipid droplet accumulation (*P* < 0.05). They upregulated LC3Ⅱ/LC3Ⅰ, Atg7, and Beclin 1 while downregulating p62. Additionally, KSJG and FF decreased p-mTOR and p-ULK1 (Ser757) but increased p-AMPK and p-ULK1 (Ser555),while Compound C could reverses this.

**Conclusion:**

OP exhibited a negative correlation with lipid metabolism indexes of NAFLD. Bilateral ovariectomy (OVX) simultaneously induced OP and NAFLD, while KSJG granules ameliorated lipid dysregulation via autophagy modulation. KSJG granules enhanced autophagy activation through the AMPK/ULK1 (Ser555) pathway, thereby alleviating NAFLD-associated lipid metabolic disturbances.

## 1 Introduction

Non-alcoholic fatty liver disease (NAFLD) is a metabolic condition defined by abnormal lipid deposition in hepatocytes, developing in the absence of significant alcohol intake or other identifiable liver injury (Pafili and Roden, 2021). Currently, NAFLD is regarded as the most prevalent chronic condition, threatening the health of nearly 30% of the global population (Huh et al., 2022). The global prevalence of NAFLD increases with age (Koehler et al., 2012). Aging exacerbates insulin resistance, obesity, and chronic inflammation, forming a vicious cycle of “aging-metabolic disorder-liver injury” (Guo et al., 2022). Research shows that aging profoundly affects the occurrence and development of NAFLD through cellular functional decline, mitochondrial abnormalities and systemic metabolic disorders (Li et al., 2023).

Osteoporosis (OP) is a metabolic bone disorder. Its primary pathological features include reduced bone mass and damaged bone microstructure, leading to decreased bone density, heightened bone fragility, and increased fracture risk (Anthamatten and Parish, 2019). According to statistics, over 200 million people worldwide are affected by OP (Lin et al., 2024). A study has shown notable gender disparities in the incidence of osteoporosis, highlighting that women aged 50 and above exhibit a four times higher incidence rate than men, and women typically develop fractures 5–10 years before men (Akbar et al., 2025). With aging, osteoblast activity decreases while osteoclast function increases, disrupting the equilibrium between bone formation and resorption. The decrease in estrogen levels, especially after menopause in women, will further accelerate bone loss (Baggio et al., 2013).

Both bone and liver are active endocrine organs with diverse metabolic functions. Metabolic syndrome (MS) is a well-established contributor to NAFLD development, with NAFLD being regarded as the hepatic manifestation of MS. Furthermore, studies suggest that MS patients may face an increased risk of BMD (Diao et al., 2023; Fan et al., 2025). NAFLD patients are often with chronic low-grade inflammation. These pro-inflammatory factors can act on bones through the bloodstream, inhibit osteogenic differentiation and activate osteoclasts, leading to a decrease in bone density (Zhao et al., 2023). At the same time, the pathological and physiological connections between NAFLD and OP are not fully understood, making it particularly important to understand the molecular pathways and mechanisms linking these two diseases (Montaseri et al., 2020).

Research indicates that autophagy regulates both bone remodeling and hepatic lipid metabolism (Pierrefite-Carle et al., 2015; Wang et al., 2023; He et al., 2024). By influencing the activity of osteoblasts, osteocytes, and osteoclasts, autophagy helps maintain bone homeostasis. Autophagy dysregulation can disrupt bone homeostasis, thereby leading to OP (Zhao et al., 2023; Yin et al., 2019; Li et al., 2020). It exerts dual regulatory functions in hepatocyte metabolism. When nutrients are insufficient, autophagy regulates the degradation of lipid droplets in hepatocytes, breaking them down to produce free fatty acids (FFA) and ATP, providing energy for the body (Raza et al., 2023); when over-nutrition leads to fat accumulation, autophagy is inhibited to prevent excessive lipid accumulation in the body (Feng et al., 2023).

Chinese medicine thinks liver kidney homology. The kidney is connected with the liver through the bone marrow, forming a “mother-child” relationship. Therefore, the liver and the kidney influence and cooperate with each other physiologically. The KSJG granules are compound formulas that have been optimized and improved based on clinical experience. The main metabolites of this formula include *Epimedium brevicornu* Maxim. [Berberidaceae; Epimedii folium], *Eucommia ulmoides* Oliv. [Eucommiaceae; Eucommiae cortex], *Achyranthes bidentata* Blume [Amaranthaceae; Achyranthis bidentatae radix], *Atractylodes macrocephala* Koidz. [Asteraceae; Atractylodis macrocephalae rhizoma], and *Salvia miltiorrhiza* Bunge [Lamiaceae; Salviae miltiorrhizae radix et rhizoma]. Our study innovatively proposes that autophagy may serve as the potential biological pathway underlying the “liver kidney homology” theory. Specifically, the formula activates hepatic autophagy pathway to ameliorate lipid metabolic disorders. At the same time, it inhibits the autophagy pathway in bone tissue and promotes osteoblast autophagy.

Both NAFLD and OP have become globally prevalent metabolic diseases, posing a serious threat to public health and economic development. There is a significant correlation between the epidemiological trends and pathogenesis of these two diseases. Previous research has confirmed that KSJG granules can effectively improve OP through the autophagy pathway. Based on the Chinese medicine theory, it is hypothesized whether KSJG granules can also have therapeutic effects on NAFLD. To verify this hypothesis, this study analyzed the correlation between OP and NAFLD using clinical data, established *in vivo* and *in vitro* NAFLD models through ovariectomy (OVX) and high-fat induction methods. To explore the effect and mechanism of KSJG granules on NAFLD under the same mechanism of improving OP. To provide richer clinical evidence for the correlation between OP and NAFLD, and to offer new insights into the treatment pathways for NAFLD.

## 2 Materials and methods

### 2.1 Clinical samples

The study recruited 261 eligible adult women from the Affiliated Hospital of Shaanxi University of Traditional Chinese Medicine in Xianyang, Shaanxi Province, China, between January 1 and March 31, 2025. Inclusion criteria: (1) women ≥18 years old; (2) completion of dual-energy X-ray for BMD; (3) availability of complete fasting lipid profile result. Exclusion criteria: (1) severe primary cardiovascular, hepatic, pulmonary, renal, or hematological disorders; (2) secondary conditions affecting bone or lipid metabolism; (3) history of OP therapy; (4) current use of lipid-lowering agent.

The clinical indicators observed in this study as follows: (1) BMD: The bone density of the L1-L4 vertebrae and the proximal end of the left femur of the patient was scanned. In clinical diagnostic protocols, the T-score serves as a fundamental parameter for diagnosing OP. The T-score is determined by the formula: (BMD of the patient - mean BMD of healthy young people)/ standard deviation of the reference population. (2) BMI Index: Measure the height (cm) and weight (kg) of each patient, and then calculate the body mass index (BMI) for each patient. BMI is an important indicator in assessing overweight and obesity status. BMI was determined using the standard calculation: BMI = weight (kg)/height^2^ (m^2^). (3) Biochemical Index: The primary biochemical indicators, encompassing blood lipids and liver function. These indicators included TC, TG, LDL-C, HDL-C, ALT, AST, total bilirubin, and direct bilirubin.

### 2.2 Animals and drugs

*In vivo* experiments used Sprague-Dawley (SD) rats (female, 3–4 months old, 200–250 g, SPF grade) provided by the Experimental Animal Center of Air Force Medical University (SCXK 2023–001). The rats were housed in an SPF-grade room with a 12-h light and dark cycle at 25 °C. During the feeding period, animals had free access to food and water. The KSJG granules (provided by the Institute of Basic Research and New Drug Development at Shaanxi University of Chinese Medicine). These granules were stored at 4 °C after high-temperature sealing (1 g of granules is equivalent to 10.15 g of crude drug) and were diluted with distilled water to various concentrations as required for the experiment. The formulation consists of five standardized botanical drugs: *Epimedium brevicornu* Maxim (15 g), *Eucommia ulmoides* Oliv (12 g), *Achyranthes bidentata* Blume (12 g), *Atractylodes macrocephala* Koidz (20 g), and *Salvia miltiorrhiza* Bunge (10 g). Process optimization was achieved through orthogonal testing (L9 (3^4) design) and response surface methodology, yielding the following validated parameters: aqueous extraction with 10-fold water volume (three extractions, 90 min each) and ethanolic extraction with 8-fold 70% ethanol (two extractions, 120 min each). Granulation employed starch: lactose (3:2) as excipients (drug:excipient ratio 1:2) with 80% ethanol as the binding agent.

In accordance with the requirements of the ConPhyMP statement, high-performance liquid chromatography, thin-layer chromatography, and ultraviolet spectrophotometry were used to qualitatively and quantitatively analyze the medicinal materials, as detailed in our prior work (Li et al., 2016; Shi et al., 2015; Du et al., 2015; Du et al., 2014). The preparation complies with the standards of the Chinese Pharmacopoeia (2020 Edition), with each gram containing no less than 1.68 mg of ICA. The medicinal materials were identified by Shaanxi University of Chinese Medicine and preserved in its Herbarium of Chinese Medicinal Materials. Icariin (ICA) (Purchased from Sichuan New Green Pharmaceutical Technology Development Co., Ltd.).

### 2.3 Establishment of the NAFLD model and grouping

A total of 60 female SD rats were randomly allocated into six groups according to their body weight: control group, model group, low-, medium-, and high-dose intervention groups of KSJG granules and ICA group. One week after adaptive feeding, OVX was performed on all rats in the groups except the control group, in which only an equal amount of adipose tissue near the bilateral ovaries was removed. Other treatments were consistent among all groups. Three months later, 3 rats from both the control and model groups were randomly selected, anesthetized, and euthanized. Liver tissues were collected for H&E staining and TG level analysis to verify successful establishment of the NAFLD model.

### 2.4 Dosage and administration

Administer the drug 1-week post-surgery. The dosage for the intervention group was calculated based on the equivalent dose relationship between humans and rats (6.5 g/70 kg), according to the Reagan-Shaw formula, this human dose was multiplied by a conversion factor of 6.2 to obtain the equivalent medium dose for rats: 0.573 g/kg/d. ICA (icariin), as the primary active metabolite of KSJG, was selected as a positive control to specifically evaluate whether the KSJG demonstrates superior therapeutic effects compared to its single active metabolite. The ICA group referencing the medium-dose group to ensure equitable comparison and eliminate potential confounding from dose-dependent effects. Specifically, the control group (normal saline/d), model group (normal saline/d), low-dose KSJG group (0.287 g/kg/d), medium-dose KSJG group (0.573 g/kg/d), high-dose KSJG group (1.146 g/kg/d), and ICA group (0.573 g/kg/d). The gastric administration volume is 0.5 mL/100 g (1 time/day, 6 times/week for 3 months).

After 3 months of intragastric treatment, blood samples were obtained via abdominal aorta. Liver tissues were harvested, with one portion fixed in 4% paraformaldehyde for H&E staining and the remainder frozen at −80 °C along with serum for subsequent studies.

### 2.5 Measurement of body weight, liver wet weight and liver index

The body weight of rats was measured weekly to adjust the intragastric administration dose. After euthanasia, the liver was immediately excised and weighed, and the liver index (liver wet weight/ body weight × 100%) was calculated.

### 2.6 H&E staining

After the liver was fixed with 4% paraformaldehyde, and then embedded in paraffin wax and sliced with a thickness of 4 μm; dewaxed to water, then stained with HE staining, observed under the microscope.

### 2.7 Western blot

70 mg of liver tissue was subjected to high-throughput grinding. Then centrifuged (4 °C, 14000 g/min, 20 min) and collected the middle layer liquid. Added the lysis buffer to treat the tissue homogenate and extracted the tissue protein. Protein concentrations were determined via BCA assay, and samples were denatured in loading buffer (100 °C, 5 min). Following cooling, samples were preserved at −80 °C. Electrophoresis was performed in a vertical chamber (120V, 60 min) using pre-cast separation and stacking gels. Subsequently, proteins were transferred to PVDF membranes, which were then blocked with 5% skim milk (2 h, room temperature). Subsequently, membranes were incubated with appropriately diluted primary antibodies at 4 °C overnight under gentle agitation. The next day, they were washed three times with TBST buffer (5 min per wash). For secondary antibody incubation, the membranes were immersed in 5 mL of 5% skim milk solution containing 1 μL of secondary antibody and incubated at room temperature for 2 h, followed by four TBST washes (5 min each). Finally, protein bands were visualized using a gel documentation system and quantitatively analyzed with ImageJ software.

### 2.8 Cell culture

Human liver L02 cells were obtained from the Laboratory of Health Toxicology, Air Force Medical University. Cells were maintained in high-glucose DMEM containing 10% fetal bovine serum (FBS) and 1% penicillin-streptomycin antibiotic mixture, incubated at 37 °C in a humidified 5% CO_2_ atmosphere.

### 2.9 Cell grouping and treatment

Normal L02 cells at the log-growth stage were cultured in 6 or 12-well plates. After 24 h adhesion, cells were divided into BSA (Bovine Serum Albumin, Thermo Fisher Scientific, United States of America) and FFA groups. The FFA group received 400 μM FFA (oleic acid: palmitic acid = 2:1), while the BSA group received an equal dose. After 7 days intervention, cells were collected for analysis.

Fenofibrate (FF) served as the positive control. Cells were divided into four groups: BSA, FFA, FFA + KSJG, and FFA + FF. Drug-containing serum concentration and intervention time were consistent for both traditional Chinese and Western medicine. After intervention, samples from all groups were collected for analysis.

Compound C inhibited the AMPK signaling pathway. Cells were divided into six groups: BSA, FFA, FFA + Compound C, FFA + KSJG, FFA + FF, and FFA + KSJG + Compound C. After intervention, samples from all groups were collected for further analysis.

### 2.10 Oil Red O staining

After incubation, Human liver L02 cells were subjected to Oil Red O staining kit (Servicebio, China), and the results were as follows: observed under a microscope.

### 2.11 BODIPY staining

The cover slips were cleaned and placed in a 6-well plate. Cells (1 × 10^4^ per well) were suspended in 1 mL complete medium and incubated at 37 °C with 5% CO_2_. Post-adherence, cells were fixed, fluorescently labeled, and imaged.

### 2.12 Dose screening of compound C

The CCK-8 assay measured cell viability following 18 h of exposure to varying Compound C concentrations (1–10 μM). WB analysis confirmed its inhibition of p-AMPK protein and identified the optimal concentration.

### 2.13 Determination of autophagy proteins

WB was used to detect autophagy marker proteins (LC3, p62, Atg7 and Beclin1) and p-AMPK pathway proteins (Akt, mTOR, AMPK and ULK1).

### 2.14 Statistical analysis

Statistical analyses were conducted in SPSS 27.0 (IBM) and visualizations generated with GraphPad Prism 10.1.2. Categorical variables underwent χ^2^analysis. For normally distributed quantitative data and homogeneity of variance. Two-group comparisons used Student’s t-test. Multi-group comparisons employed one-way ANOVA with Least Significant Difference (LSD) *post hoc* testing. Non-parametric Kruskal–Wallis testing addressed non-normal/heteroscedastic data. Pearson correlation analysis was performed to assess the relation of NAFLD and OP. Multivariate binary Logistic regression was applied for multivariate analysis, with results as *OR* with 95% Confidence Interval (CI). *P* < 0.05 was considered significant.

## 3 Results

### 3.1 Clinical research results

#### 3.1.1 The association between general characteristics, hepatic lipid metabolism indictors, and OP

The general data and liver lipid metabolism indicators of OP group (n = 123) and non-OP group (n = 138) were compared. It was observed that the prevalence of OP increased with age (*P* < 0.01); the weight and BMI of the OP group were higher than those of the non-OP group, while the height was lower than that of the non-OP group, and the differences reached statistical significance (*P* < 0.01); the TC and TG levels in the OP group were significantly higher than those in the non-OP group (*P* < 0.01). No statistically significant differences were observed in HDL-C, LDL-C, ALT, AST, total bilirubin, and direct bilirubin between the two groups (Table 1).

**TABLE 1:** Comparison of general data between OP group and non-OP group ( $\bar{x}\pm s$ ).

Variable	OP (n = 123)	Non-OP (n = 138)	t/χ^2^	*P*
age (y) n (%)			45.9	<0.001
<50	1 (4.5)	21 (95.5)		
50–59	28 (31.5)	61 (68.5)		
60–69	31 (54.4)	26 (45.6)		
70–79	47 (62.7)	28 (37.3)		
≥80	16 (88.9)	2 (11.1)		
Weight (kg)	58.3 ± 7.2	53.2 ± 6.6	5.97	<0.001
Height (cm)	151.9 ± 5.7	155.1 ± 4.6	5.01	<0.001
BMI	25.3 ± 3.0	22.1 ± 2.5	9.40	<0.001
BMD (g/cm^2^)	0.6 ± 0.1	0.8 ± 0.1	16.13	<0.001
Z-score	−1.3 ± 0.8	0.1 ± 1.2	10.95	<0.001
T-score	−3.5 ± 0.8	−1.1 ± 1.1	19.95	<0.001
TC (mmol/L)	5.8 ± 1.5	4.9 ± 1.0	5.76	<0.001
TG (mmol/L)	2.1 ± 1.1	1.4 ± 0.7	6.20	<0.001
HDL-C (mmol/L)	1.3 ± 0.4	1.3 ± 0.3	0.00	>0.999
LDL-C (mmol/L)	3.2 ± 0.9	3.1 ± 0.9	0.90	0.371
ALT (U/L)	21.7 ± 24.7	23.1 ± 14.8	0.56	0.574
AST (U/L)	26.1 ± 17.8	24.4 ± 14.1	0.86	0.391
Total bilirubin (umol/L)	15.2 ± 8.4	14.0 ± 7.2	1.24	0.215
Direct bilirubin (umol/L)	5.6 ± 4.7	5.0 ± 3.1	1.23	0.220

#### 3.1.2 Correlation analysis between BMD, T-score and hepatic biochemical indexes

We assessed potential associations using Pearson’s correlation coefficients to quantify linear relationships between BMD and T-scores, and age, BMI, TG, TC, HDL-C, LDL-C, ALT, AST, total bilirubin, and direct bilirubin. The results indicated that BMD and T-scores were correlated with age, BMI, TC, TG, and ALT. Specifically, BMD exhibited a significant negative correlation with age, weight, TG, and TC, while showing a positive correlation with ALT (*P* < 0.05). Moreover, T-scores were significantly negatively correlated with LDL-C (*P* < 0.05).

**FIGURE 1:**
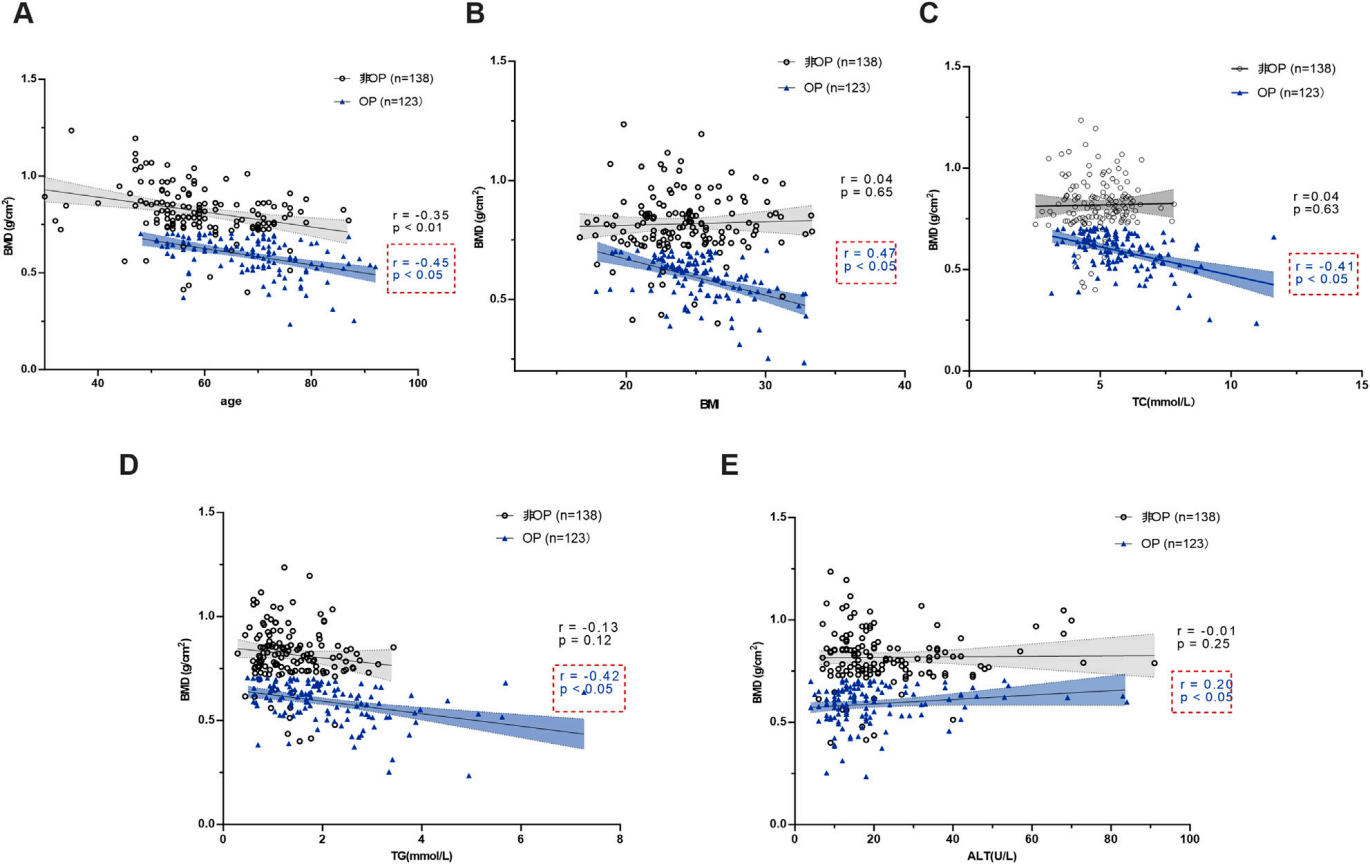
Correlation analysis of BMD between the two groups and age, BMI, TC, TG and ALT. **(A)** BMD and age. **(B)** BMD and BMI. **(C)** BMD and TC. **(D)** BMD and TG. **(E)** BMD and ALT.

Stratified analysis revealed that BMD values in both groups were significantly negatively correlated with age (*P* < 0.05). In the OP group, BMD values showed significant negative correlations with BMI, TC, and TG, and a significant positive correlation with ALT (*P* < 0.05). By contrast, no correlations were observed between BMD values and the above indices in the non-OP group (Figure 1).

#### 3.1.3 Multivariate analysis of the effects on OP

The results showed that age (β = −0.103, OR = 0.902, *P* = 0.001), BMI (β = −0.535, OR = 0.585, *P* = 0.000), TC (β = −0.617, OR = 0.539, *P* = 0.033) and TG (β = −0.623, OR = 0.536, *P* = 0.031) were risk factors for OP (Table 2).

**TABLE 2:** Multivariate analysis of factors affecting OP.

Variable	β	S. E	Wald	*P*	*OR*	95% CI	
						Lower	Upper
age (y)	−0.103	0.020	26.406	0.001	0.902	0.867	0.938
BMI	−0.535	0.081	43.352	0.000	0.585	0.499	0.687
TC (mmol/L)	−0.617	0.290	4.524	0.033	0.539	0.305	0.953
TG (mmol/L)	−0.623	0.288	4.666	0.031	0.536	0.305	0.944
HDL-C (mmol/L)	0.152	0.662	0.052	0.819	1.164	0.318	4.257
LDL-C (mmol/L)	0.631	0.349	3.269	0.071	1.880	0.948	3.726
ALT (U/L)	0.019	0.015	1.605	0.205	1.020	0.989	1.051
AST (U/L)	−0.025	0.018	1.814	0.178	0.976	0.942	1.011
Total bilirubin (μmol/L)	0.009	0.048	0.036	0.850	1.009	0.918	1.110
Direct bilirubin (μmol/L)	0.022	0.109	0.042	0.838	1.023	0.825	1.267

### 3.2 *In vivo* experimental results

#### 3.2.1 The improvement effect of KSJG granules on liver injury in NAFLD

Relative to control animals, model group rats exhibited dry and yellow fur, obesity, and reduced activity and responsiveness. Their livers were significantly enlarged, appearing yellow, with a rough texture and oily surface.

Prior to modeling, no significant differences in body weight were observed among the groups. During the modeling and drug intervention phases, body weight increased in all groups. The model group demonstrated the most substantial increase in body weight, with statistically significant differences emerging from the second week (*P* < 0.05). Treatment with NAFLD and ICA, effectively alleviated the rise in body weight (*P* < 0.05).

Relative to control animals, model group rats showed higher liver wet weight and liver index. However, all other treatment groups effectively reduced these parameters, with the high-dose group exhibiting the most pronounced effect.

HE staining showed the liver cells in the model group were swollen, some cells were balloon-like, the nuclei were pushed to one side, and many liver cells contained lipid droplet vacuoles of different sizes and quantities. Relative to control group, the model group exhibited significantly elevated TG levels (*P* < 0.05). The degree of fatty degeneration of liver cells in the low-, medium-, and high-dose groups, as well as the ICA group, was significantly improved. Among them, the high-dose group had the most significant reduction effect.

**FIGURE 2:**
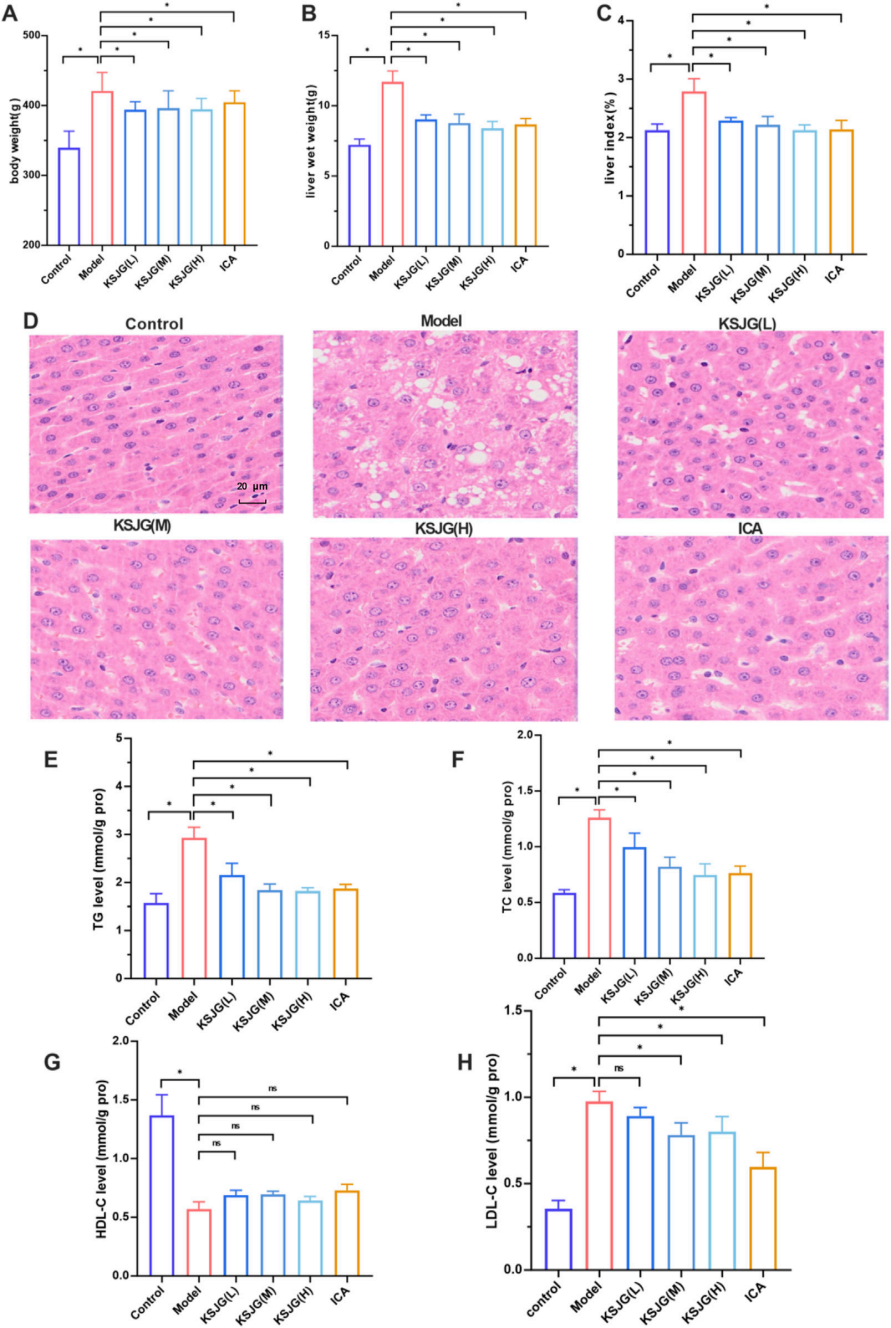
Effects of KSJG granules on rat liver. **(A)** Body weight. **(B)** liver wet weight. **(C)** liver index. **(D)** Representative photomicrographs of liver tissue (H&E staining, 400 X). **(E–H)** Hepatic lipid profiles: TG, TC, HDL-C, and LDL-C levels. *P < 0.05 vs. Model group.

In the model group, TG, TC, and LDL-C levels increased, while HDL-C levels decreased (*P* < 0.05). In the low-, medium-, and high-dose group and the ICA groups, TG, TC, and LDL-C levels decreased (*P* < 0.05) (Figure 2).

#### 3.2.2 The effect of KSJG granules on autophagy proteins and pathways`

The model group exhibited significantly reduced protein expression of autophagy markers (LC3Ⅱ/LC3Ⅰ, Atg7, and Beclin1), accompanied by elevated p62 levels (*P* < 0.05). The medium-dose, high-dose and ICA groups could all increase the expression levels of LC3Ⅱ/LC3Ⅰ, Atg7 and Beclin1 proteins to varying degrees and decrease the p62 level, with the medium-dose group showing the most significant effect (*P* < 0.05). Among them, the low-dose group increased the LC3Ⅱ/LC3Ⅰ level and decreased the p62 level, but there was no statistically significant difference in Atg7 and Beclin1.

**FIGURE 3:**
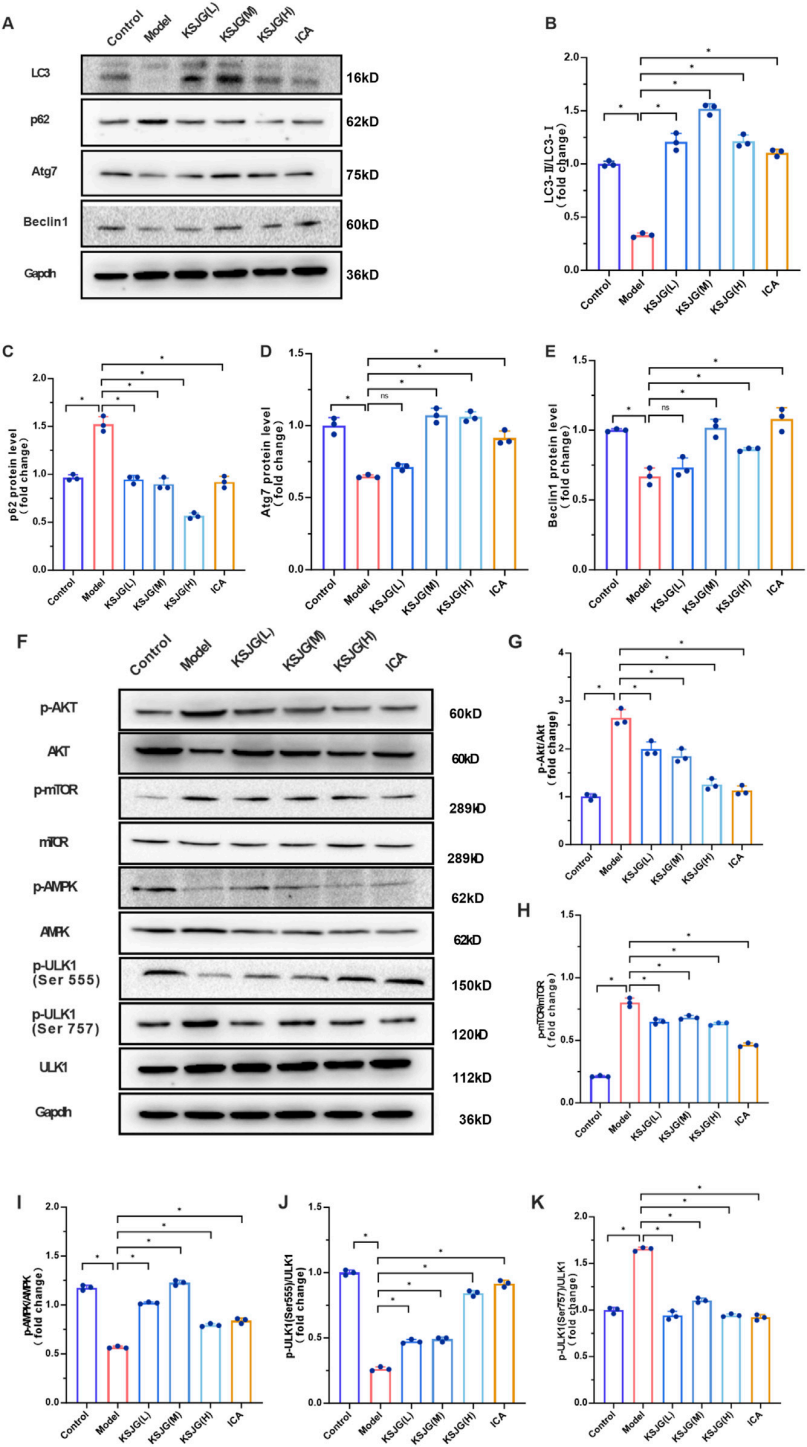
Effects of KSJG granules on autophagy-related proteins in rat liver. **(A)** Representative Western blot bands of autophagy markers. **(B–E)** Quantitative analysis of LC3II/LC3I ratio, p62, Atg7, and Beclin1 protein levels. **(F)** Western blot bands of autophagy pathway proteins. **(G–K)** Quantification of p-Akt, p-mTOR, p-AMPK, p-ULK1 (Ser555), and p-ULK1 (Ser757) protein expression. *P < 0.05 vs. Model group.

Relative to the control group, the expression levels of p-Akt, p-mTOR, and p-ULK1 (Ser757) proteins were elevated in the model group, while the levels of p-AMPK and p-ULK1 (Ser555) decreased (*P* < 0.05). The low-, medium-, and high-dose and ICA groups, could reduce the expression levels of p-Akt, p-mTOR, and p-ULK1 (Ser757) to varying degrees, and increase the levels of p-AMPK and p-ULK1 (Ser555) (*P* < 0.05). Among these, the high-dose group showed the most significant differences in reducing p-Akt and p-mTOR levels and increasing p-ULK1 (Ser555) levels, while the medium-dose group had the most significant effect in increasing p-AMPK levels (*P* < 0.05, Figure 3).

### 3.3 *In vitro* experimental results

#### 3.3.1 Establishment of L02 high lipid cell model

**FIGURE 4:**
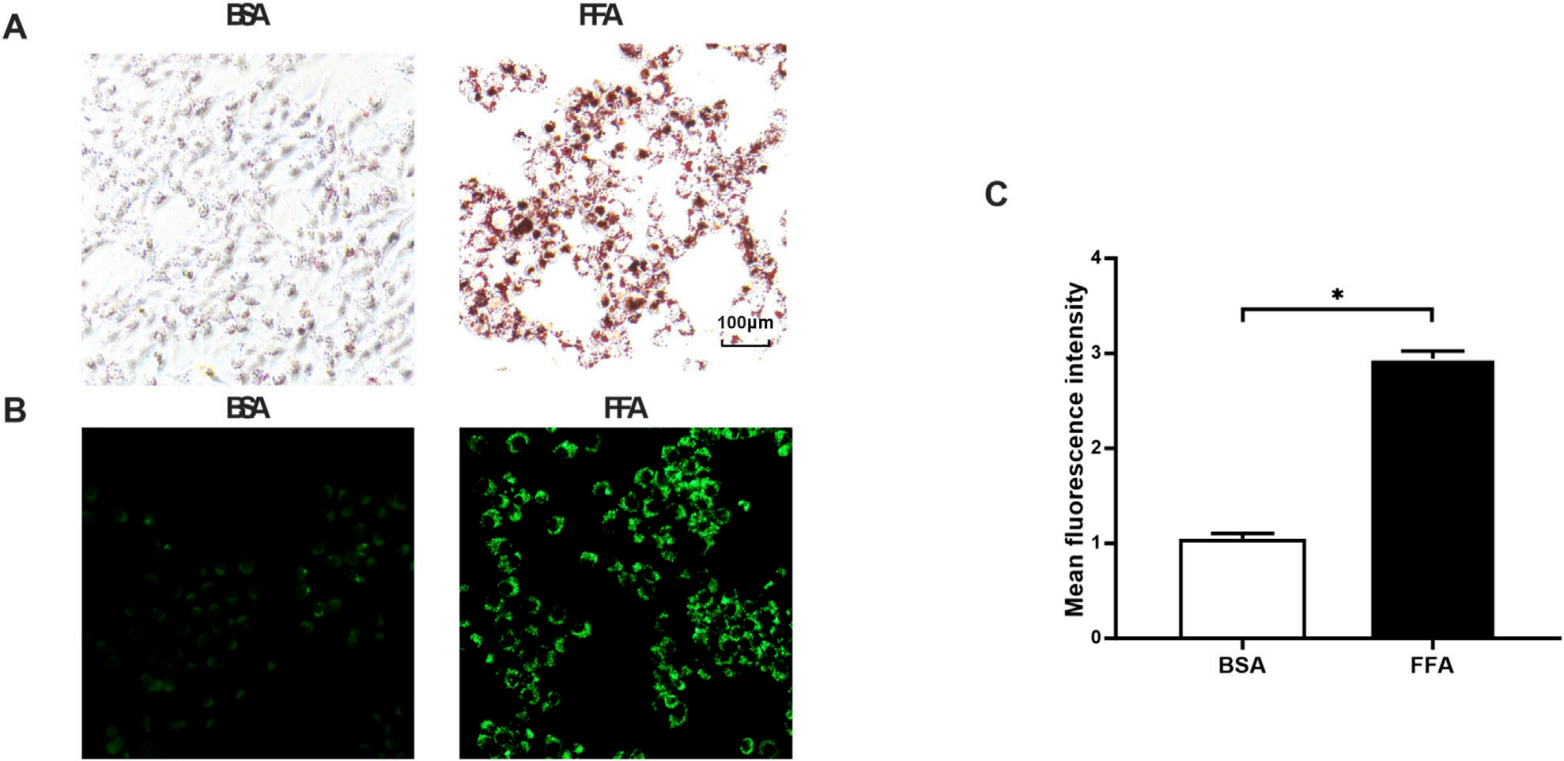
Effects of FFA on lipid accumulation in L02 cells. **(A)** Oil Red O staining (200 x). **(B)** BODIPY staining (200 x). **(C)** Quantitative analysis of BODIPY fluorescence intensity. *P < 0.05 vs. FF group.

Oil Red O staining revealed markedly increased lipid accumulation in FFA-treated cells relative to the BSA control, with significantly more red-stained droplets observed. This was further confirmed by BODIPY 493/503 staining, where FFA-exposed cells exhibited enhanced green fluorescence intensity corresponding to lipid droplets. These consistent findings demonstrate that FFA effectively induces intracellular lipid deposition, successfully establishing a cellular model of steatosis (Figure 4).

#### 3.3.2 KSJG granules alleviate lipid accumulation in high-fat cells and hepatocyte injury

The CCK8 method was used to detect the effects of different drug-containing serum concentrations on cell viability in the FFA group. The results showed that under 0%, 10%, 20%, 30%, 40%, and 50% drug-containing serum concentrations, there were no statistically significant differences in cell viability. However, when 10% drug-containing serum was used, the cell viability was relatively better. Therefore, 10% drug-containing serum was selected for experiments.

The Reitman-Frankel method kit was used to detect the effects of different intervention times on FFA group cell ALT levels in 10% drug-containing serum. The results showed that compared to the FFA group, cell ALT levels were significantly reduced at 8 h, 12 h, 16 h, 20 h, and 24 h intervention times, with the most significant effect observed at 8 h, indicating the best efficacy (*P* < 0.05). Therefore, 8 h was selected as the intervention time for subsequent experiments.

**FIGURE 5:**
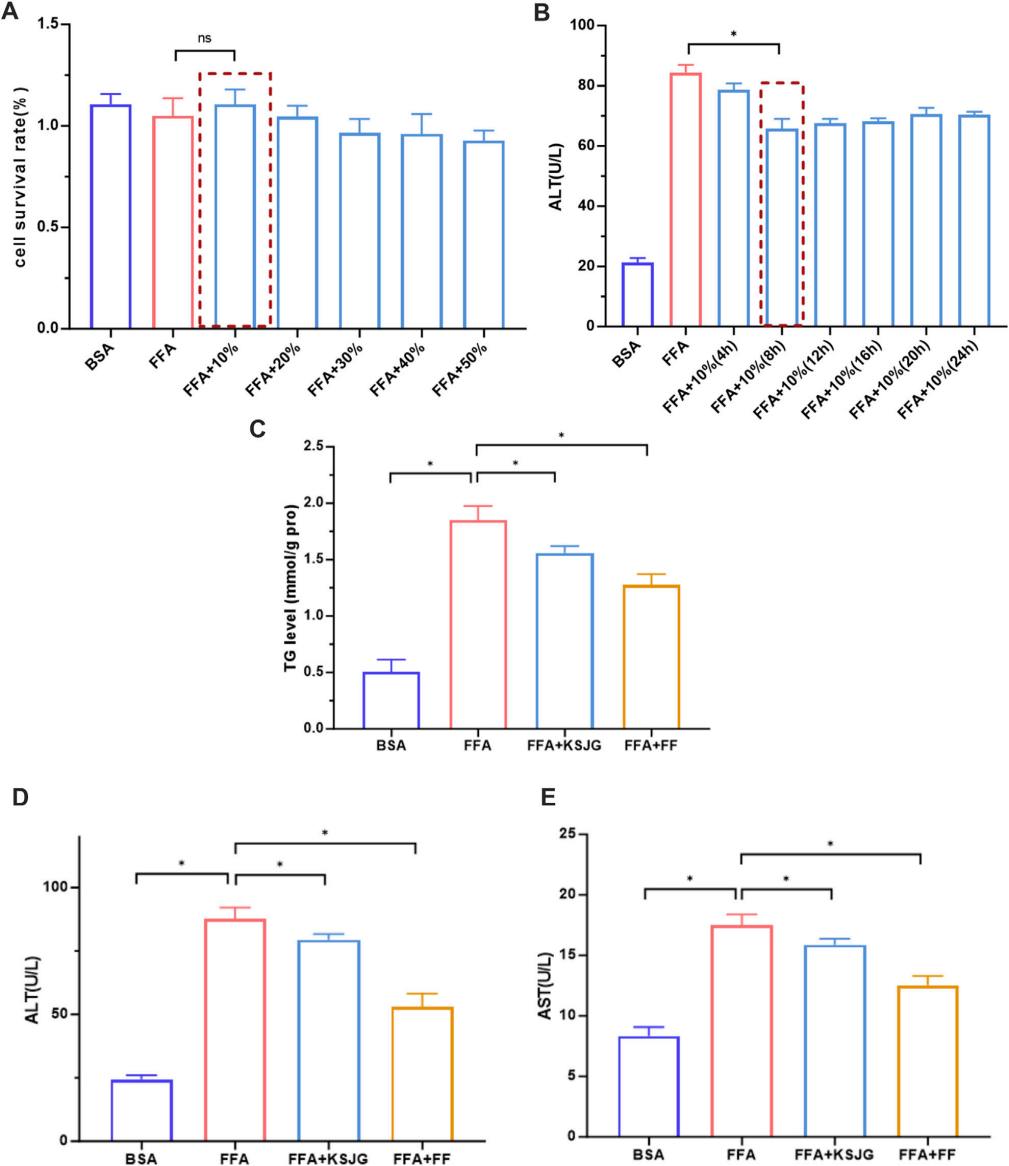
Effects of KSJG granules on lipid metabolism and hepatocyte injury markers. **(A,B)** Screening of serum concentration and action time containing drug (*P < 0.05 vs. FFA); **(C–E)** Effects of drug-containing serum on TG, ALT and AST in cells (*P < 0.05 vs. FFA).

Elevated TG levels are one of the key manifestations of NAFLD. The TG level test results showed that, compared to the BSA group, the FFA group had significantly higher TG levels, while both the FFA + KSJG and FFA + FF groups significantly reduced TG levels (*P* < 0.05). ALT and AST are important indicators of acute hepatocyte injury and have high sensitivity to hepatocyte damage. The FFA group has significantly higher ALT and AST values, while the FFA + KSJG and the FFA + FF groups effectively reduced the ALT and AST levels (*P* < 0.05). These results indicated that KSJG granules can effectively improve lipid accumulation in high-fat cells and hepatocyte injury, providing a certain degree of protection for hepatocytes (Figure 5).

#### 3.3.3 The effects of KSJG granules on lipid accumulation under AMPK-inhibited conditions

The impact of Compound C (1–10 μM) on cell survival was quantitatively measured via CCK-8 methodology. The cell viability under the intervention of 1–4 μM Compound C had no statistically significant difference from that of the control group. Therefore, the concentration range of Compound C was set at 1–4 μM.

The protein level of p-AMPK was detected by WB at 1–4 μM Compound C after intervention. The 4 μM Compound C treatment group showed maximal suppression of p-AMPK expression relative to control group (*P* < 0.05). Therefore, 4 μM Compound C was selected for AMPK inhibitor in subsequent experiments.

**FIGURE 6:**
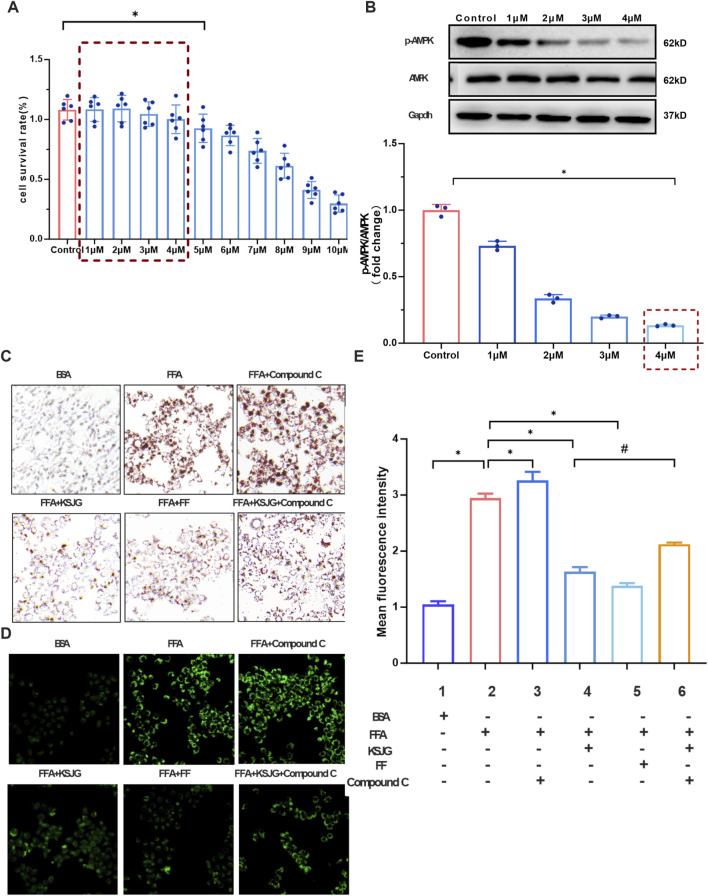
The effects on liver cell metabolism and signaling pathways. **(A)** Cell viability assessed by CCK-8 assay. **(B)** p-AMPK protein band and quantification (*P < 0.05 vs. control group). **(C)** Oil Red O staining of lipid accumulation (200X). **(D)** BODIPY staining of lipid droplets (200X). **(E)** Quantification of BODIPY fluorescence intensity (*P < 0.05 vs. FFA group, ^#^ *P* < 0.05 vs. FFA + KSJG).

Quantitative analysis of both Oil Red O and BODIPY 493/503 staining revealed a significant increase in LD formation and intracellular lipid accumulation in the FFA group compared to BSA group (*P* < 0.05). The lipid accumulation in the FFA + Compound C group further increased, while the LD content in the FFA + KSJG and the FFA + FF groups significantly decreased (*P* < 0.05). Adding Compound C to the FFA + KSJG group could inhibit the KSJG effect, leading to an increase in LD content (*P* < 0.05). The above results indicated that the inhibition of AMPK exacerbated FFA-induced lipid accumulation in cells, while the drug-containing serum of KSJG granules improved the level of lipid accumulation induced by FFA after AMPK was inhibited (Figure 6).

#### 3.3.4 The effects of KSJG on autophagy-related proteins and pathways under AMPK-inhibited conditions

Protein quantification demonstrated that FFA treatment downregulated autophagy markers (LC3Ⅱ/LC3Ⅰ, Atg7, Beclin1) while upregulating p62 accumulation relative to the BSA group (*P* < 0.05). Relative to the FFA group, the levels of LC3Ⅱ/LC3Ⅰ, Atg7 and Beclin1 in the FFA + Compound C group were further decreased, and the level of p62 was increased. However, the FFA + KSJG and the FFA + FF groups could significantly increase the levels of LC3Ⅱ/LC3Ⅰ, Atg7 and Beclin1, and decrease the level of p62 (*P* < 0.05), thereby enhancing autophagy. Additionally, adding Compound C to the FFA + KSJG group could reverse the effect of KSJG (*P* < 0.05), inhibit autophagy, but there was no statistically significant difference in Beclin1.

**FIGURE 7:**
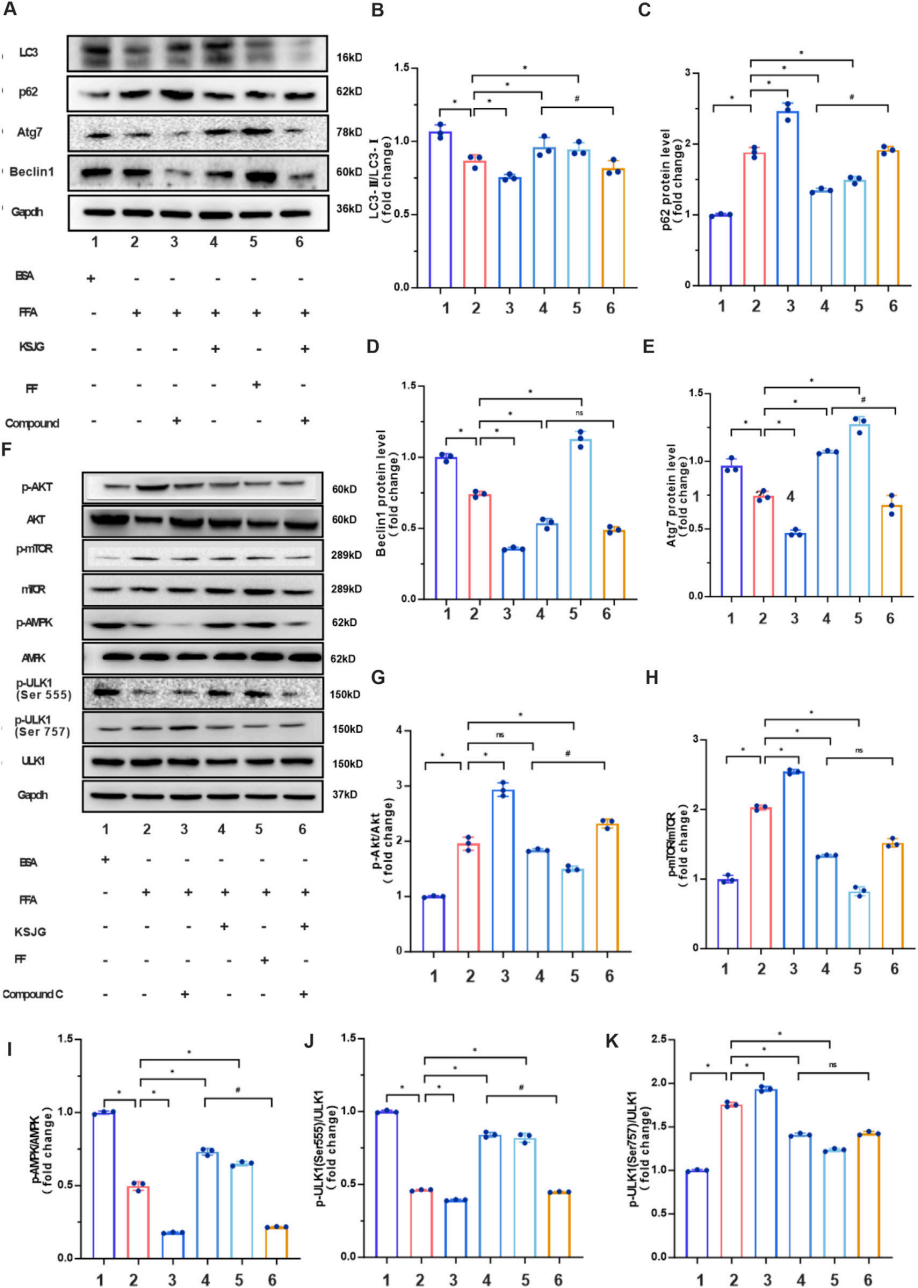
KSJG granules regulate autophagy in Compound C treated models. **(A)** Representative Western blot images of autophagy markers. **(B–E)** Quantitative analysis of LC3II/LC3I ratio, p62, Atg7, and Beclin1 protein expression (*P < 0.05 vs. FFA group; ^#^ *P* < 0.05 vs. FFA + KSJG group). **(F)** Western blot analysis of autophagy pathway proteins. **(G–K)** Quantification of p-Akt, p-mTOR, p-AMPK, p-ULK1 (Ser555), and p-ULK1 (Ser757) phosphorylation levels (*P < 0.05 vs. model group).

We quantified Akt, mTOR, AMPK, and ULK1 protein expression across all six experimental groups using WB analysis. The results demonstrated that, the FFA group exhibited increased expression of p-Akt, p-mTOR, and p-ULK1 (Ser757), while the levels of p-AMPK and p-ULK1 (Ser555) were significantly reduced (*P* < 0.05). The FFA + Compound C group further enhanced the protein expression of p-Akt, p-mTOR, and p-ULK1 (Ser757) to varying extents and decreased the levels of p-AMPK and p-ULK1 (Ser555). Conversely, the FFA + KSJG and FFA + FF groups markedly suppressed the protein expression of p-mTOR and p-ULK1 (Ser757) and promoted the levels of p-AMPK and p-ULK1 (Ser555) (*P* < 0.05), with no significant alteration observed in p-Akt levels. Notably, the addition of Compound C to the FFA + KSJG group partially reversed the effects of KSJG on these proteins (*P* < 0.05), although no significant differences were detected in p-mTOR and p-ULK1 (Ser757) levels (Figure 7).

## 4 Discussion

As two closely related metabolic diseases, OP and NAFLD have significant connections. Research indicates that both are associated with abnormal differentiation of bone marrow mesenchymal stem cells (osteoblasts and adipocytes) and share common risk factors (obesity and dyslipidemia) (An et al., 2023). Lipid metabolism disorder affects bone metabolism, and obesity and metabolic disorders facilitate the initiation and advancement of NAFLD (Yuan et al., 2023). Research has shown that obesity-related indicators, such as BMI, TG, TC, and HDL-C, can serve as predictors of NAFLD (Xie et al., 2021). Our team found that KSJG granules can improve OP by regulating autophagy (Wang et al., 2021). This study hypothesizes whether KSJG granules can also have preventive and therapeutic effects on NAFLD. To verify this hypothesis, the research is conducted through three parts: clinical, *in vivo*, and *in vitro*.

Clinical research systematically analyzed the correlations between OP and lipid metabolism-related indicators, as well as the risk factors of OP. Our analysis revealed significant correlations between OP and several metabolic parameters, including age, body mass index, total cholesterol, and triglyceride levels. BMD is an important index to assess bone mass and bone strength. However, T-score is usually used as the gold standard for diagnosing OP in clinical practice. T-score is a relative value reflecting the difference between BMD of the patient and normal young people (Mäkitie and Zillikens, 2022). The correlation analysis was conducted on the BMD, T-scores, age, BMI, TG, TC, HDL-C, LDL-C, ALT, AST, total bilirubin, and direct bilirubin of patients included in the study. The results indicate that age is closely related to BMD values. In addition, the BMI, TC and TG of OP patients re negatively correlated with BMD.

Through binary Logistic regression analysis, it was found that age, BMI, TC and TG were risk factors for OP. Studies have confirmed the association between OP and NAFLD, with age being a significant influencing factor. As age increases, endocrine function declined, bone metabolism and lipid metabolism become disordered, and the rate of bone remodeling decreases, making it easier to develop OP (Farr and Khosla, 2019). Similarly, aging exacerbates liver injury through metabolic dysregulation. BMI is an important indicator of obesity, which has a negative impact on bone metabolism. This negative impact may be caused by changes in the bone micro-environment and systemic inflammation. The formation of NAFLD is mainly closely related to lipid metabolism disorders, manifested as an increase in fat synthesis in liver cells and a decrease in oxidation, especially the imbalance in the synthesis and metabolism of TC and TG (Amor and Perea, 2019). The above results indicate that the aging process mediates the interplay between OP and NAFLD pathogenesis. OP is significantly associated with abnormal lipid metabolism, but the interaction and mechanism between them remain unclear.

OVX is a recognized method for the establishment of OP models. In our study, we found that NAFLD can also be established at the same time as OP. *In vivo* studies employed the OVX rat model under the same mechanism of OP to observe lipid metabolism changes and the effect of KSJG granules on NAFLD lipid metabolism. HE staining and TG level detection proved the establishment of the NAFLD model.

Traditional Chinese medicine treatment emphasizes the principle of “syndrome differentiation and individualized therapy”. For patients with NAFLD, different treatment strategies are adopted based on their distinct clinical symptoms and stages of disease progression. Owing to its diverse therapeutic approaches and high safety profile, it has garnered increasing attention in clinical practice. KSJG granules represent an empirical formula that has been continuously refined and optimized through extensive clinical experience (Shi et al., 2016).

Metabolic disorders in the body are important factors leading to obesity and liver lipid accumulation (Fahed et al., 2022). Further experiments found that KSJG granules effectively controlled body weight, reduced liver wet weight and liver index; improved lipid accumulation and lipid metabolism disorder. These results demonstrate that KSJG granules exert estrogen-like effects to regulate metabolism, thereby reducing hepatic lipid accumulation.

Autophagic dysfunction as a key pathogenic driver in NAFLD progression. Long-term high-sugar and high-fat diets generate excessive fatty acids, leading to a sustained increase in free fatty acids in the body. This leads to the long-term accumulation of lipid droplets within cells, thereby reducing the autophagy level of liver cells (Ren et al., 2024). Impaired autophagosome-lysosome fusion disrupts hepatic TG β-oxidation, resulting in lipid droplet accumulation and contributing to NAFLD pathogenesis (Zhou et al., 2022). The KSJG granules increased the expression levels of LC3II/LC3I, Atg7 and Beclin1 proteins and decreased the level of p62, indicating that the KSJG granules could improve the autophagy level of NAFLD rats through the same mechanism as OP.

As an energy sensor in cells, AMPK serves as a key upstream regulator of autophagic processes, activating autophagy through multiple signaling pathways. ULK1 serves as the core regulatory that initiates autophagosome formation, and activated AMPK can promote autophagy by directly or indirectly acting on different sites of ULK1 (Feng et al., 2024). When AMPK is activated, the central integrator of the mTOR signaling pathway and growth factors can inhibit mTOR phosphorylation through multiple pathways, thereby regulating cellular glucose metabolism, lipid metabolism. mTOR is a negative regulator of autophagy. Its activation can lead to the phosphorylation of the downstream substrate ULK1 (Ser757), thereby negatively regulating autophagy. Additionally, AMPK can directly act on ULK1 (Ser555) to positively regulate autophagy (Alers et al., 2012). Akt and AMPK are both upstream signaling factors of mTOR, which can activate mTORC1 and reduce the level of autophagy. The KSJG granules increased autophagy level in liver tissue, reduced the expression of p-Akt, p-mTOR and p-ULK1 (Ser757), and increased the expression of p-AMPK and p-ULK1 (Ser555). These results suggest that KSJG granules enhance hepatic autophagy through AMPK pathway regulation.

To further explore whether KSJG granules inhibit lipid accumulation and lipid metabolism disorder in the liver through AMPK-dependent autophagy. *In vitro* study used FFA solution to induce lipid accumulation in human liver L02 cells to establish a NAFLD cell model. The mixture of oleic acid and palmitic acid has long been used in NAFLD cell models (Zhang et al., 2023). Fenofibrate (FF) is one of the commonly used lipid-lowering drugs in clinical practice. Multiple studies have demonstrated that FF significantly ameliorates liver injury and dyslipidemia in OVX rats (Shin et al., 2021; Mahmoudi et al., 2022). In this study, FF served as the reference compound to evaluate the efficacy of KSJG. Both KSJG granules and FF could reduce the levels of TG, ALT and AST, suggesting that KSJG granules can effectively improve lipid accumulation in high-fat cells and liver cell damage.

Compound C, a commonly used chemical inhibitor, can reduce AMPK activity (Liu et al., 2014). By using 4 μM of the AMPK inhibitor Compound C, we found that KSJG granules could promote autophagy and improve cellular lipid accumulation through the AMPK pathway. The experiments showed that after inhibiting AMPK, lipid accumulation increased, while the expression of autophagy marker proteins LC3Ⅱ/LC3I, Atg7 and Beclin1 decreased, and the expression of p62 increased. KSJG granules could significantly reverse these changes, but this effect could be counteracted by Compound C, indicating that KSJG regulates the autophagy process by activating AMPK. Further research found that KSJG granules could reduce the expression of p-mTOR and p-ULK1 (Ser757), while increasing the levels of p-AMPK and p-ULK1 (Ser555). Compound C could reverse these regulatory effects of KSJG. These results confirmed that KSJG granules regulate the level of autophagy by activating the AMPK/ULK1 (Ser555) pathway, thereby improving lipid metabolism disorders in liver cells.

The AMPK-specific activation by KSJG may be attributed to the synergistic effects of its multiple metabolites: *Epimedium brevicornu* Maxim directly phosphorylate AMPK, while *Salvia miltiorrhiza* Bunge and *Atractylodes macrocephala* Koidz inhibit key nodes in the Akt and mTOR pathways. While autophagy activators (such as rapamycin) acts through single-target mTORC1 inhibition, this approach often leads to metabolic complications such as insulin resistance caused by feedback activation of Akt signaling. However, KSJG prevents the activation of compensatory pathways (Salmon, 2015). The KSJG mechanism further differs from metformin, which indirectly activates AMPK through AMP/ATP-mediated pathways. KSJG directly phosphorylates ULK1 at Ser555, enabling more efficient initiation of autophagy. This comprehensive, multi-component approach not only enhances therapeutic efficacy but also minimizes off-target effects, establishing KSJG as a promising candidate for osteoporosis treatment with potentially fewer side effects than conventional therapies (LaMoia and Shulman, 2021).

KSJG granules demonstrates a “treat different diseases together” therapeutic potential, effectively improving OP through AMPK/ULK1 autophagy pathway activation while regulating hepatic lipid metabolism to alleviate NAFLD. Its phytoestrogenic activity additionally mitigates menopausal symptoms. Compared with conventional estrogen replacement therapy (ERT), KSJG exhibits superior safety profiles: its unique ICA metabolite selectively activates ERβ receptors to avoid ERT-associated carcinogenic risks; *Salvia miltiorrhiza* Bunge provides natural anticoagulation to eliminate thrombosis risks; while simultaneously regulating lipid metabolism to prevent common metabolic side effects of ERT, offering postmenopausal women a safer therapeutic alternative (Nikolić et al., 2017).

In conclusion, this study suggests that OP is negatively correlated with lipid metabolism indicators in NAFLD. Bilateral OVX can simultaneously cause OP and NAFLD. KSJG granules can improve lipid metabolism disorders caused by NAFLD through the same mechanism as that of OP by activating the AMPK/ULK1 (Ser555) pathway to upregulate autophagy levels. These findings suggest that KSJG granules is an effective drug for NAFLD and OP, provide a new idea of “treating different diseases with the same therapy”.

## Data Availability

The datasets presented in this study can be found in online repositories. The names of the repository/repositories and accession number(s) can be found in the article/supplementary material.

## Abbreviations

AMPK, adenosine monophosphate-activated protein kinase; Atg7, autophagy-related 7; BMD, bone mineral density; BSA, Bovine Serum Albumin; CI, Confidence Interval; ERT, estrogen replacement therapy; FFA, free fatty acid; FF, fenofibrate; ICA, Icariin; KSJG, Kangshujiangu granules; LC3, microtubule-associated protein 1 light chain 3; mTOR, mammalian target of rapamycin; MS, metabolic syndrome; NAFLD, non-alcoholic fatty liver disease; OVX, ovariectomy; OP, osteoporosis; ULK1, Unc-51-like autophagy activating kinase 1.
